# Comparison of the lived experiences of family caregivers of patients with dementia and of patients with cancer in Indonesia

**DOI:** 10.1017/S1041610217001508

**Published:** 2017-09-05

**Authors:** Martina Sinta Kristanti, Yvonne Engels, Christantie Effendy, Astuti, Adi Utarini, Myrra Vernooij-Dassen

**Affiliations:** 1 IQ Healthcare, Radboud Institute for Health Sciences, Radboud University Medical Center, Nijmegen, The Netherlands; 2 School of Nursing, Faculty of Medicine, Universitas Gadjah Mada, Yogyakarta, Indonesia; 3 Department of Anesthesiology, Pain and Palliative Care, Radboud University Medical Center, Nijmegen, The Netherlands; 4 Neurology Department, Sardjito Hospital, Yogyakarta, Indonesia; 5 Public Health and Policy Department, Faculty of Medicine, Universitas Gadjah Mada, Yogyakarta, Indonesia

**Keywords:** dementia, cancer, Indonesia, family caregiver, lived experience, social health

## Abstract

**Background:**

Dementia, even more than cancer, demands long-term care. While in Indonesia cancer is accepted as a disease requiring caregiving, dementia is still considered "a normal condition." These differences might affect the experiences of caregivers, especially those relating to social health, the subject of our study. We aim to describe and compare the lived experiences of family caregivers of patients with cancer (PWC) with those of patients with dementia (PWD) in Yogyakarta, Indonesia, and to explore the role of their social health in these experiences.

**Method:**

A qualitative design was applied. In-depth face-to-face interviews were conducted with PWC and PWD caregivers in two outpatient clinics of a tertiary hospital. The constant comparative method was applied to analyze the data that were interpreted using the concept of social health to explore the experiences of the caregivers. We used Atlas.ti software.

**Results:**

Three themes were identified: problems with caregiving, dealing with problems, and beliefs in caregiving. We found more similarities than differences in the experiences of caregivers in both groups. Half of the categories were related to social health: challenges, consequences, hiding, social support, and the caregiver's approach. The organization of dementia care is characterized by simplicity and direct ties between medical specialists, PWD, and caregivers, whereas cancer care encounters coordination problems.

**Conclusions:**

Family caregivers of both groups mostly had similar experiences of the caregiving process. Gaining a better understanding of the specific experiences of caregivers, and their social health, opens new avenues for interventions to improve their quality of life.

## Introduction

The number of non-communicable diseases (NCD) in Indonesia is escalating. Cancer, Alzheimer's, and other types of dementia are claimed to contribute increasingly to the mortality rate from NCD (Wang *et al.*, 2016). There were almost 300,000 new cases of cancer in Indonesia in 2012 (Ferlay *et al.*, 2013). In fact, it is ranked number seven among Asian countries. Meanwhile, it is predicted that in 2050, 70% of people suffering from dementia will mostly reside in the low- and middle-income countries, including in Indonesia (Wortmann, 2012). A recent survey reported that Indonesia is fourth after China, India, and Japan, with over 600,000 people with dementia compared to the whole Asia Pacific region, which has around 13 million people (Access Economics, 2006).

Cancer as well as dementia profoundly influences the well-being not only of the patients, but also of the family caregivers. The characteristics of these diseases might influence the burden of caregiving. Therefore several studies have compared the burden of family caregivers of patients with cancer (hereafter referred to in this paper as PWC) and those of persons with dementia (hereafter referred to as PWD) with various results. Some studies reveal that family caregivers of both groups experience a similar level of burden (Kim and Schulz, 2008; Costa-Requena *et al.*, 2015). Other studies show that family caregivers of PWD (hereafter referred to as FCD) have a higher level of burden (Papastavrou *et al.*, 2012) and experience more stress than those caring for PWC (hereafter referred to as FCC) (Sampson *et al.*, 2016), although one study found the opposite (Harding *et al.*, 2015). Apart from that, family caregivers of both groups experience a loss of identity (Gillies and Johnston, 2004) decreased health outcomes (Sampson *et al.*, 2016) and experienced an anticipatory grieving (Johansson *et al.*, 2013).

Caring is essentially related to the relationship between the family caregiver and the patient. Therefore, the lived experience of caregiving might also be associated with social health. Social health involves making a dynamic balance between opportunities and limitations, and is affected by external conditions such as social and environmental challenges (Huber *et al.*, 2011). Social health consists of three major dimensions including: people's capacity to fulfill their potential and obligations, the ability to manage their life and participating in their social life or work (Huber *et al.*, 2011). Regarding family caregiving, the relationship with the patient, as well as the external conditions like cultural norms and values, is important for the social health of family caregivers.

Ethnic differences appear to influence family caregivers’ outcomes, including burden and depression (Pinquart and Sorensen, 2005). In Indonesia, strong family and community bonds exist, which may affect the way family caregivers provide care for their loved ones with cancer or dementia. Indonesian people, like those in other Asian countries, perceive such caregiving tasks as a duty (ADI and Australia, 2014) that should not be questioned (Funk *et al.*, 2011). Besides, in contrast to developed countries, dementia is seen as a normal ageing process and not as a life-threatening disease (ADI and Australia, 2014; Cipriani and Borin, 2015). Family caregivers continue to involve persons with dementia as much as possible in social activities, which might contribute to the social health of persons with dementia. On the other hand, the social health of family caregivers might be influenced by the experience of such caregiving.

Getting insights into the lived experiences of FCC and FCD, and in the related role of their social health, is needed to develop new interventions that actively facilitate the utilization of their social and environmental resources (Vernooij-Dassen and Jeon, 2016). As research on caregiving is very Western-oriented (Poveda, 2003), information on the lived experiences of family caregivers in other cultures is sorely needed.

Therefore, the aim of the current study is to describe and compare the experiences of FCC with FCD in Indonesia, and to explore the role of their social health in these experiences.

## Methods

### Design

We use a qualitative design to get information about the lived experiences of FCC and FCD. The family caregivers who were invited were the spouse, adult-child, or relative who looks after a patient with any kind of cancer in stage 2–4, or looks after a PWD with a Mini-Mental State Examination (MMSE) score of ≤ 24/30. We chose family caregivers from these groups of patients, as in these stages patients may experience physical and psychological changes due to treatment and disease progression. Besides, most patients will be more or less dependent on the family caregivers if in this condition. Other inclusion criteria were being the main person who is taking care of such a patient for at least 6 months up to the date of inclusion, living with this patient or delivering the care to the patient for at least 3 hours a day. We considered duration of care and number of hours spent are important to identify family caregivers who are most involved in the caring process. Finally, we selected participants who are adults (being ≥ 18 years). They also had to be willing to take part in the study. Participants were invited via the outpatient clinics for cancer and dementia of Sardjito Hospital, Yogyakarta, Indonesia. This tertiary care hospital has more than 800 beds and has been accredited nationally as well as internationally by the Joint Commission International (JCI).

The study was approved by the Medical and Health Research Ethics Committee (MHREC), Faculty of Medicine, Universitas Gadjah Mada – Dr. Sardjito Hospital, Indonesia (KE/FK/744/EC 24 Jun 2015), and data collection was permitted by the hospital.

### Recruitment of participants

Participants were selected purposively, striving for a mixture of gender, age, relationship with the patient, job, income, level of education and religion. In this way, we received a richer variation in these demographic characteristics (Robinson, 2014) and thus in the lived experiences of FCC and FCD. The head nurse or a physician in the collaborating outpatient clinics introduced the study to potential participants. After that, the first author checked the eligibility criteria of those family caregivers who were interested. When eligible, the participants received comprehensive information about the study procedures and protocols. They were also informed that they could withdraw at any time during or after the interview without any consequences. Once a participant agreed to be part of the study, he or she signed an informed consent form.

### Data collection

Data on FCC were collected from the chemotherapy unit between July and August 2015. Data on FCD were collected from the memory clinic between July and September 2016. Based on the current literature, a topic guide was developed and reviewed by a multidisciplinary team from two countries: Indonesia and the Netherlands (each of whom will be referred to by their initials) consisting of a sociologist, who is also a professor specializing in the psychosocial aspects of dementia and palliative care (MVD); a physician and professor in public health (AU), a neurologist and dementia expert (A), an associate professor in timely palliative care (YE), an epidemiologist and expert in cancer and palliative care (CE), as well as a nursing lecturer and PhD student trained as a qualitative researcher (MSK). The interview guide consisted of five questions regarding the types of caregiving topics: a list of the tasks, experience, burden, motivation, and the positive aspects of caregiving (Table 1).

**Table 1.** Topic guide

topic	questions
List of tasks	Could you please tell us your daily activities?
	1. In relation to the patient's activities?
	2. In relations to your activities?
	3. Has the caregiving process changed your life?
Experience	Could you please tell me your experience in taking care of your loved one?
	1. How do you feel about it?
	2. Do you think it is different being a caregiver for cancer/dementia and another illness?
Burden	1. How hard is it to take care of your family?
	2. What do you find the most difficult task in the caregiving process?
	3. Which area do you think you need more support?
	4. Who supports you as a caregiver? Your community? Family?
Motivation	1. What is your main reason for taking care of your family?
	2. Do you have the feeling to do that, or do you choose to do that?
Changes in life	1. What are the changes that have occurred in your life due to your caregiving tasks?
	2. What do you learn/gain from the caregiving process?

Semi-structured, in-depth, face-to-face interviews were conducted by the first author (MSK), who had not had any contact or relationship with the participants prior to the interviews. These interviews lasted between 30 and 90 minutes each and were audiotaped. Interviews took place at the hospital, in a quiet private room. Field notes were made during and after the interviews to record non-verbal observations, which were integrated into the transcripts for data analysis. After 23 interviews, no new codes were found. Two more interviews were done to check the data's saturation.

### Data analysis

The audiotaped interviews were transcribed verbatim in the Indonesian language by an independent transcriber. The transcripts were then read line by line by the first author (MSK) to check their accuracy. Transcripts were then read line by line for coding development, using Atlas.ti 7th edition software. Coding was done in English to facilitate the discussion with all the authors. These codes were developed from meaningful words, phrases or statements in the transcripts. Several coding development sessions with the Indonesian and Dutch authors were arranged to make sure that the codes had a comparable, suitable, and similar meaning in Indonesian and English. Also, the final book of codes was discussed in a meeting with all authors. The constant comparative method was used to analyze the data (Glaser, 1965). Data collection and data analysis were performed in parallel sessions and continued until no new codes were found. With this method, the codes derived from the previous interviews were the starting point for coding the next transcript; new codes could then be added or the codes merged if needed. Next, with the adapted list of codes, the previous transcripts were re-read, as suggested by Glaser (1965). This entire process continued after each interview until no new codes were found.

At the beginning of the process, two authors (MSK and CE) read the transcripts of the interviews of the FCC and started the open coding process by coding them independently. Subsequently, these codes were discussed and mutually compared, seeking any similarities and discrepancies with two other authors (AU and MVD) until a consensus was reached. Once the open coding was completed, grouping into categories took place in several sessions with all the authors until consensus was again reached.

Next, the second round of data collection with FCD was conducted; transcripts of these interviews were analyzed using the book of codes developed from the transcripts of the interviews with FCC. Data analysis with a constant comparative method allowed us to develop coding inductively (Glaser, 1965); therefore, the book of codes from the previous round in FCC was used and new codes for FCD data were added where needed. Another session for categorization development was held with all the authors. Next, themes were developed from the categories. Finally, social health, following the definition of Huber *et al*. (2011), was used to interpret the data. Consolidated criteria for reporting qualitative research (COREQ) was used to report the study's result.

## Results

Of the 32 family caregivers approached, seven refused to participate: Three FCC because of their patients’ condition, and four FCD due to time restraints or because they lived far away. In total, 25 family caregivers were interviewed: 13 FCC and 12 FCD. About half of the participants (52%) were female and 60% were spouses. FCC (41.8 years) as well as the patients they cared for (53.7 years) were younger than FCD (59.9 years) and patients with dementia (69.1 years) (Tables 2 and 3).

**Table 2.** Characteristics of family caregivers of patients with cancer (FCC)

			relationship	patient's	patient's	patient's
code	gender	age	with patient	gender	age	cancer type
P1	Male	62	Husband	Female	60	Breast
P2	Male	55	Husband	Female	59	NPC
P3	Male	25	Son	Female	54	Breast
P4	Male	47	Husband	Female	42	Breast with malignant wound
P5	Female	46	Sister	Female	59	Ovarian
P6	Female	39	Daughter	Female	59	Ovarian
P7	Female	31	Daughter	Male	72	Larynx
P8	Female	50	Wife	Male	56	NHL
P9	Male	50	Husband	Female	51	Ovarian
P10	Female	46	Daughter	Male	77	Sigmoid
P11	Female	24	Daughter	Male	50	Melanoma
P12	Male	31	Brother	Male	35	NHL
P13	Female	43	Wife	Male	44	Rectal with malignant wound

**Table 3.** Characteristics of family caregivers of patients with dementia (FCD)

			relationship	patient's	patient's	patient's
code	gender	age	with patient	gender	age	mmse score
P14	Female	69	Wife	Male	80	10
P15	Male	59	Husband	Female	54	10
P16	Male	40	Son-in-law	Female	73	20
P17	Male	65	Husband	Female	61	20
P18	Female	58	Wife	Male	67	20
P19	Male	70	Husband	Female	71	23
P20	Female	65	Wife	Male	73	24
P21	Male	63	Husband	Female	54	20
P22	Female	40	Daughter-in-law	Female	81	24
P23	Female	54	Daughter	Female	87	10
P24	Male	66	Husband	Female	61	20
P25	Female	62	Wife	Male	65	23

Three main themes were identified: problems in caregiving, dealing with problems, and beliefs regarding caregiving; each containing several categories (Table 4). Many categories were derived from both groups, and some from just one. Additionally, within some categories, a part of the codes was only derived from one group.

**Table 4.** Codes, categories, and themes for the lived experiences of FCC and FCD

exist in cancer (C) and or dementia (D)	key codes	category	theme
C	Long and complicated administrative procedures, workload of the staff, communication and coordination with health professionals, need for support from healthcare professionals	Quality of service	Problems in caregiving
C	Financial burden, financial support/sharing, insurance process and benefits.	Financial aspects	
C + D	Patient's personality, psychological changes in the patient, distance from hospital, conflicts, quality and quantity of information, changes in patients’ clinical aspects, practical matters, expectations for caregiver, longing for relationship	Challenges	
C + D	Multiple tasks, multiple roles, adding activities, commuting, time spent, physical burden, another family to care for, reduced income, don't receive certain support, emotional impacts.	Consequences	
C	Avoid crying and confrontation, pretend to be strong, things not to be discussed, not discussing diagnoses.	Hiding	Dealing with issues
C + D	Acceptance, balancing life, adaptation of one's life to caregiving, continuing activities, flight, blaming, proactive in seeking information, hopes, trial and error	Coping mechanism	
C + D	Support from others, flexibility at one's workplace, several caregivers in the house, sharing the caregiving task, community as informal guards	Social support	
C + D	Contributions from several family members, process of decision making for the patient, patient is the main priority, keep the patient happy, planning for the future.	Approach of family caregiver	
C + D	Reasons for caregiving: Voluntary, obligatory	Motivation to care	Beliefs in caregiving
C + D	Values in life, perceptions about disease and treatment, spiritualism in disease and treatment	Faith	
C + D	Changes in personal life, extra benefits/advantages for self, staying positive, and family cohesiveness.	Positive changes in personal life	

### Description of family caregivers’ experiences

#### Theme 1: Problems in caregiving

This theme reflects the issues faced by family caregivers. Four categories have been identified: Quality of service, financial aspects, challenges, and consequences.

##### Quality of service

This category reflects the critical comments regarding the healthcare institutions. Family caregivers reported that they needed to learn and understand some unfamiliar and complicated procedures. There were long queues for administrative procedures, while the quality and length of time they spent with healthcare staff was limited. Family caregivers also expected to have a friendlier and more empathic contact with the healthcare staff than they actually experienced.

##### Financial issues

This category illustrates their financial situation during treatment. Most family caregivers received some funding from National Insurance to cover the patients’ treatments. This was very helpful and highly appreciated. Although most basic treatments are covered by insurance, there are some other expenses that could not be covered such as specific diagnostic procedures, certain specific drugs, travel costs, and other expenses like food and accommodation for caregivers when treatment occurred in a hospital far from their home. With regard to the financial issues, some family caregivers reported being in debt as a result of covering their patient's treatments.

##### Challenges

This category concerns situations the family caregivers had to deal with during the caregiving process. The first challenge came from their own concerns about the quality of the care they provide, not from their patient's requests. They worried about problems such as their patient's physical changes or nutrition, and how to cope with them. Participants also stated that conflicts with other family members escalated their burden. These conflicts related to different opinions about the practical arrangements for the caregiving, or the ignorance of other family members. Their patient's behavioral changes are also a challenge for the family caregivers. Finally, some family caregivers, especially spouses, revealed that they longed for the relationship they previously had, as their patients became different persons due to their disease's progress.

##### Consequences

Consequences reflect the unpleasant physical or emotional effects of caregiving, which were most likely due to the challenges faced. Family caregivers reported having additional activities, like doing domestic chores or taking over the patient's tasks, which affected them physically. They reported feeling tired and exhausted. Some participants stated that they also needed to dress or care for wounds at home, for which they had not had any instruction. Other consequences were the changes in their role, such as taking over the patient's position as the breadwinner, and having to face multiple roles. Negative consequences were also caused by the denial of the situation by other family members. Feelings of loneliness were ascribed to spending most of their time with their patient, which reduced their usual social activities. Some family caregivers revealed that they felt overburdened, and suffered from a lack of concentration and experience a loss of interest in things. Family caregivers also expressed that they felt powerless due to the whole situation.

#### Theme 2: dealing with problems

This theme concerns solving problems faced during the caregiving process. Four categories emerged from this theme: coping mechanisms, approach to caregiving, hiding, and social support.

##### Coping mechanisms

These mechanisms reflect problem-solving strategies that allow the caregivers to deal with upcoming issues. The participants used some constructive coping mechanisms. Family caregivers used social coping by sharing their stories. Most family caregivers reported using spiritual coping to ease their problems, including being more religious while in their current situation. Proactively seeking information was also reported by family caregivers, who joined a community for cancer or dementia carers, using the internet and finding written information. Family caregivers also coped by maintaining hope, balancing their life by trying to continue their social activities and by catharsis activities to release their stress. Family caregivers reported that accepting their situation helped them to cope with it. However, some family caregivers reported less constructive ways of coping, such as the flight mechanism, which ignores new information about their patient's condition, and by spreading blame onto other people and things. Some family caregivers also blamed themselves for having contributed to their patient's condition. Others blamed the patient for not being cooperative, or the healthcare staff for their style of communication or service provision.

##### Approach of the family caregiver

The strategies for care and treatment include decision making, sharing caregiving tasks, sharing the financial burden, and complying with the wishes of the patient. Patients, whenever possible, appeared to be part of the decision-making process. Other family members and relatives, especially those with medical or healthcare systems’ knowledge, were also involved in this process. Especially, if the patient was a parent, the children shared the caregiving tasks. Adult children contributed in any way they could. Some family caregivers arranged a schedule for the division of caregiving. They also shared the financial burden; sometimes the patient was unaware of such financial arrangements. Most family caregivers considered meeting the patient's wishes as their highest priority; keeping the patient happy was a part of their task. Due to the patients’ cognitive impairment, some family caregivers found communicating with them difficult.

##### Hiding

This strategy concerns the family caregivers’ behavior to suppress their real emotions toward their patient's condition or their situation. Participants avoided showing the truth and their real emotions, like tears and sadness, in front of the patients. Certain issues, like death and future care planning, were not discussed within the family or with the patient. Also, a family sometimes concealed the diagnosis from the patient, especially at the beginning of the process. The family waited for the right moment to disclose it. Some of them did not disclose it at all, especially when it concerns their elders.

##### Social support

This category reflects any kind of available assistance from their social networks. Some family caregivers reported having another family member with whom they are able to share the caregiving task. There was some meaningful indirect support given by social networks, which was appreciated by the family caregivers. This kind of support was sometimes not directly given to the patient, but it lifted the caregivers’ burden. For example, blessings from the husbands to perform caregiving for their parents or siblings were considered important, as mentioned by several women in this study. Support was also received from the carers’ workplaces. They received permission for their absence or were granted more flexible working hours. Receiving social support from their local community also reduced their challenges. Some participants felt blessed that their neighbors or friends were willing to watch their children or provide meals for them while they were away caregiving.

#### Theme 3: beliefs related to caregiving

This theme reflects the family caregivers’ willpower to perform caregiving tasks. Three categories emerged: the motivation to care, faith, and positive changes in the caregiver's personal life.

##### Motivation to care

This particular category identifies the reasons for the family caregivers taking up the caregiving task. Most family caregivers voluntarily chose to provide care. They insisted on being part of the caring journey. A few of the participants thought that it was an obligatory duty and resented the caregiving task. One participant felt he was forced into caregiving, as no one in his family was willing to provide care for his brother and he had no other choice. Some participants revealed that they were willing to take on the caregiving duty because they were chosen by their patients. Another motivation, especially in cancer care, was valuing the time spent with their loved ones, although they had to reduce their own work and productivity, which may have decreased their income. They tried to cherish every moment as they realized that they had limited time together.

##### Faith

This category describes basic principles consisting of values, perceptions, and spirituality related to the disease and its treatment. These are mostly related to religion, which often strengthened their motivation. Values about caregiving mostly concerned their belief in being chosen by God for the role of a caregiver. Some of them also had a strong belief that they or their children would receive some kind of beneficial payback by doing a good thing for others. Also, most spouses believed that the patient is their soul mate; therefore, they wanted to be in charge of the caregiving task. Participants believed that taking good care of their parents or parents-in-law would earn them a good place in heaven. A few participants believed that disease is a kind of punishment from God for some mistakes the patient made in the past. By accepting the condition and keeping praying, the patients’ sins could be purified and the patient could become spiritually "clean" again. Most of them reported that they spent more time than usual practicing their religion and became much closer to God after their patients became ill.

##### Positive changes in personal life

Despite the problems, the participants identified positive changes in their personal life. They mentioned that being a caregiver adds value to their personal lives. They felt much better as a person. Being a caregiver stimulated them to gather knowledge about the disease and the administrative processes in the hospital. They also revealed that they tend to have a healthier lifestyle. Almost all the participants reported that they experienced family cohesiveness due to their patient's disease. When they had a sick parent, the adult children would have more frequent communication with their other siblings, to share the news and update the patients’ condition, or to provide support to each other. One participant said that since they had a sick parent, the family, consisting of nine adult children, decided to meet regularly on Friday nights to pray together and update each other with the news about their parent's condition.

### Comparison of the lived experience of FCC and FCD

In general, most categories applied to both FCC and FCD; the major exceptions were in the first and second themes (Table 4). From the current study, only FCC reported having problems related to the quality of service and financial aspects. These included critical comments on the healthcare system and the financial burden due to the patient's treatments.

Due to their patients’ cognitive impairment, only FCD reported a longing to have a meaningful relationship with their patients.

I can't talk to her like in the old days. When I miss her, I just hug her tightly. Thank God, she is not complaining when I am doing that although she doesn't recognize me as her husband anymore. She can't recognize anyone anymore. [FCD-P14, Husband]

In the second theme: *Dealing with problems*, only FCC reported hiding, in which they tried to hide their emotions and certain issues from their patients. Especially, at the beginning of the process, concealing a cancer diagnosis from the patient also sometimes occurred.

Before surgery he only knew that there was a tumor in his neck. Then he asked why he needed an operation. I only told him that the doctor said that if he did not have that, he wouldn't be able to talk anymore. He may know now or have guessed that he has cancer, I don't know. [FCC-P7, Daughter]

In the category of coping mechanisms, both groups used blaming, but with different focuses. Some FCD blamed themselves for having contributed to their patients’ conditions. One participant mentioned he had worked in a different city for more than 20 years of their married life, so that the patient had to look after their children on her own during that time. He believed that this put too large a burden on her and contributed greatly to her dementia. Others blamed the patient for not being cooperative or, especially for FCC, the healthcare staff for their style of communication or service provision.

Meanwhile, only FCD considered communication to be difficult, due to the patient's cognitive impairment. FCD tended to follow their own feelings and used some tricks to fulfill their patient's needs.

I did that by trial and error, especially because I was not too close to her in the past, so I did not know her routines at the beginning. It is very hard to ask her to eat, she always refuses. I found out that she is very much concerned about not wanting to be a burden for somebody else. Then one day I tried one trick, I said: 'Mom, you have to eat . . . because if you don't eat then you will get sick, and when you get sick then you would be a burden to us and your children’ I said that . . . and it worked! I am using that trick all the time now! [FCD-P22, Daughter-in-law]

Also, only FCD mentioned that they intentionally shared the patient's diagnosis with their neighbors and community in order to get their support. In that way they made the neighbors informal guardians for their loved ones.

Yes of course I told my neighbors, it is important. Well, at least they can pray for my husband's health. Besides, I don't want them to think that he is out of his mind, he's just having dementia. [FCD-P25, Wife]

FCD reported that they had direct and intense communications with their physicians. They revealed that this good quality communication with the physicians reduced their pressure/stress. The physicians became their sources of information as well as their strength and motivation.

Even if when she [a neurologist] is out of the country, she always replies to my text-messages. I feel safe. We are grateful to have her. [FCD-P19, Husband]

We have never taken mom to the hospital, whenever we need, I text him [a geriatrist], then he would come over and check mom's condition. [FCD-P22, Daughter-in-law]

### Role of social health in the family caregivers’ lived experiences

The next step in our study was to analyze the lived experiences through the lens of social health. Thereby, we identified categories that reflected social challenges affecting the balance of opportunities and limitations for family caregivers. Five out of eleven categories were identified: challenges, consequences, hiding, social support and approaches used by the caregiver. Challenges develop during contact with the patient and other family members. Consequences concern the emotional and physical impact of caregiving. Family caregivers hid their real emotions in front of their patients, and avoided confrontations when possible. Findings, in the current study especially, describe the social networks’ support. The approach of the caregiver includes sharing their tasks with other family members and focusing on keeping the patient comfortable.

The lived experiences of caregivers appear to be strongly connected to their social health and relate both to challenges caused by the patient as well as to positive experiences.

## Discussion

By exploring the lived experiences of FCC and FCD, three themes were identified: (1) problems in caregiving, (2) dealing with problems, and (3) beliefs related to caregiving. Although caring for the family is part of the Asian culture, family caregivers generally volunteer to accept this task. We found more similarities than differences in the lived experiences between FCC and FCD. Five out of the eleven categories relate to experiences of both groups of family caregivers. The quality of service, financial aspects, and hiding of emotions, however, were only derived from FCC. Half of the categories are linked to social health. These include challenges, consequences, hiding, social support, and the approach of the caregiver. We analyzed the current literature related to the comparison of caregiving for dementia and cancer patients. Unfortunately, all studies are from developed countries, because of the lack of similar research in Asian countries.

In the theme *problems in caregiving*, family caregivers from both groups experienced challenges in relationships. FCD had the feeling of losing the relationship with their loved one for FCD due to the cognitive impairment (Kim and Schulz, 2008; Costa-Requena *et al.*, 2015). Some types of cancer can influence the quality of the relationship, especially when couples are confronted with prostate cancer (Ramsey *et al.*, 2013) or breast cancer (Ahmad *et al.*, 2015). Although several of the FCC in our study often hid their emotions and avoided confrontation, they did not experience a decreased quality in their relationship with their loved ones. In fact, FCC reported that its quality improved; some of them became emotionally closer, and highly valued this new togetherness.

Only FCC reported problems with the quality of the service especially regarding integration of care. A possible explanation might be that FCD have direct and intense communication with geriatrists and neurologists. Also, only FCC complained on financial issues. It is inconsistent with a previous study, which revealed that the financial hardship was not significantly associated with the type of disease (Kim and Schulz, 2008). Further, a study from the USA reported that in comparison to cancer and patients with other chronic diseases, FCD have the highest financial burden (Kelley *et al.*, 2015). In the current study, FCC spent more on PWC's medicine, in comparison to FCD. In regard to financial issues in cancer care, low-income countries have more financial burden and receive less government support in comparison to high income ones (Souza *et al.*, 2016). Therefore, some actions need to be implemented in order to minimize inequalities in cancer care in developing countries (Farmer *et al.*, 2010).

In the second theme: *dealing with problems*, family caregivers focused on the patients’ needs and happiness, putting their patients’ needs as their first priority. In order to achieve this goal, family members worked together and contributed as much as possible throughout the caregiving process. Family caregivers used constructive coping mechanisms such as proactively seeking information, using religion, acceptance, adapting, and balancing life. They also reported ineffective coping mechanisms, like flight and blaming.

*Hiding*, which illustrates that family caregivers suppress their emotions and sometimes conceal the diagnosis, especially at the beginning of the process, was only revealed by FCC. This finding is in line with a previous study by Gillies and Johnston (2004) and another Asian studies (Back and Huak, 2005). They argue that FCC do not want to add to the burden on the patient (Gillies and Johnston, 2004). In contrast, Asians perceive dementia as a less threatening disease (Li and Loke, 2013; Alzheimer Disease International and Alzheimer's Australia, 2014). Similarly, FCD in the current study did not find the need to conceal dementia diagnosis to patients.

In the third theme, *beliefs in caregiving*, caregivers from both groups had similar experiences. The most frequently mentioned reason to provide care was the value placed on the relationship; it made the family caregivers voluntarily accept this task. Family caregivers reported having certain values in their lives that support their caregiving tasks. Religion plays a major role in faith. The motivation to care and faith have hardly been explored before in an Asian context.

As for positive changes in their personal lives, we identified some benefits for family caregivers related to their caregiving tasks, including greater family cohesiveness and the opportunity to do good things with their lives. A previous review also reported positive changes in family caregivers’ personal life and how they receive a sense of achievement for doing caregiving tasks (Lloyd *et al.*, 2016).

### The role of culture in family caregiving

Our findings are not only an expression of the lived experiences of a caregiver for patients with a specific disease, but also of caregiving in a specific cultural context. Ethnic differences and culture contribute to the variety in caregiving process and caregivers’ outcomes (Pinquart and Sorensen, 2005). This is one of the first Asian studies comparing family caregivers in cancer and dementia.

In our Indonesian study, some specific words such as *obligation* and *calling* (meaning an intrinsic passion) were frequently used to express family caregivers’ reasons to take up a caregiving task. Family caregivers were embedded with a natural tendency to be a caregiver for their ill member of the family. In this part of the world, religion is used as a protective factor to provide comfort and support (Lim *et al.*, 2011). Similarly, participants in our study linked *obligation* and *calling* with religion. Indonesian people considered religion as one of the major parts of their life and as an important element of their identity. They believed that good things they were doing during their life will determine if they would be able to live in heaven or not. As they pursued heaven, being a caregiver would grant them a great reward. Therefore, most family caregivers were highly motivated in order to "save their place" in heaven.

Our findings are consistent with other studies that found that Asians perceive dementia as a part of the normal aging process (Li and Loke, 2013; Alzheimer's Disease International and Alzheimer's Australia, 2014). Therefore, PWD in Asia are encouraged to embrace their life in the community as normally as possible. For instance, in our study, some PWD were still highly involved in community activities, such as in weekly religious meetings or sports. Attending such activities preserved the PWD's dignity and self-esteem. FCD also found this as an appreciation for their loved one being accepted in the community. For FCD, this was also beneficial because they were able to have a little break. Western society's emphasis is on pathology; people are also stigmatized due to their cognitive impairment (Groen-van de Ven *et al.*, 2016). This fact might discourage PWD to participate in the communities’ social life (Gillies and Johnston, 2004) and gradually reduce their participation in the decision-making process (Groen-van de Ven *et al.*, 2016).

### Social health of family caregivers

Almost half of the categories appeared to be associated with social health. Those are challenges, consequences, hiding, the approach of the caregiver, and social support. They all reflect the interconnection between the family caregiver and the care recipient, and the way caregiving influences the balance between the opportunities and limitations for the family caregiver. On the one hand, this has negative physical and emotional consequences for the caregiver, to the extent that the caregiver is in need of social support, and reduces his/her activities to little other than caregiving. On the other hand, there might be reciprocity (Vernooij-Dassen *et al.*, 2011), meaning that the caregiver gets something back from the care recipient, such as a feeling of complying with existing norms and religious rules on taking care of family members, as well as perceiving better family cohesiveness, similar to what we found in our study.

### Strengths and limitations

The qualitative methods used in the current study provide the possibility to explore the rich information about the lived experience of family caregivers including problems concerning how to handle it and beliefs toward it. These areas have been rarely explored in previous comparative studies, which all used a quantitative approach. The current study used the same topic guide for both groups of family caregivers, which facilitated a direct comparison of their experiences. Both groups of family caregivers were from the same region, which also facilitated the comparison. The use of the COREQ was beneficial in reporting these complex data (Tong *et al.*, 2007).

Social health of family caregivers is an innovative concept that we attempted to elaborate through the current study. One of the limitations is that the interview guidelines used in the current study were developed to explore more general aspects of the lived experience of family caregivers, rather than just focusing on social health issues. Finally, although several meetings with all authors for coding development were arranged, in the translation process, some cultural-sensitive information might have been lost.

### Implication for research and practice

The richness of the findings in the current research has generated new research questions such as how caregivers influence the social health of their patients and about the role of culture in caregiving.

The results of dealing with dementia often contrast with those of Western studies. This contrast relates to the basic assumptions concerning the dementia diagnosis and its consequences: seeing dementia as a part of the normal ageing process or using a focus on pathology. Both approaches prevent blaming the patient and blaming either ageing or pathology. The Asian approach allows the patients "continuity of normality," while the Western approach seems to induce exclusion from their wider social environment. Also, the discrepancy in the appreciation of the quality of care between FCC and FCD highlights the effects of complex care. While cancer care is rapidly developing in Indonesia and suffers from coordination problems, dementia care is characterized by its simplicity and the direct ties between medical specialists, patients, and family caregivers, as well as the wider community; FCD perceive these direct ties as being very beneficial. The implications of these approaches should also be considered with an eye for the advantages and disadvantages in each of them, with the intention to learn from each one.

A future study should focus on potential interventions to involve the family caregiver as well as the community in the caregiving process. For example, because most participants are willing to provide care at home, home-based support might be a better option than setting up hospice care. As religion appears to strongly influence coping mechanisms and the family's perceptions on disease and treatment, this area, which affects social health, may need to be taken into account in such interventions. Also, although family caregivers from both groups seem to have more similarities than differences, a future study may focus on identifying the problems and needs of family caregivers in each group. A better connection with the specific lived experiences of family caregivers, and to social health, opens new avenues to interventions to improve their quality of life.

## Conflict of interest

None.

## Description of authors’ roles

M. Sinta Kristanti is a PhD candidate who designed the study, conducted the interviews, conducted data analysis, and wrote the paper. Astuti and C. Effendy contributed in selecting and identifying the participants as well as supervised data collection process. M. Vernooij-Dassen, A. Utarini, Y. Engels, and C. Effendy are the supervisors. All authors contributed to the data analysis. All contributed and approved the final version of the paper.
